# A Tribute to Professor Andrew Otis Jackson

**DOI:** 10.3390/v18010056

**Published:** 2025-12-30

**Authors:** Fangfang Li, Jonathan Griffiths, Xueping Zhou, Aiming Wang

**Affiliations:** 1 State Key Laboratory for Biology of Plant Diseases and Insect Pests, Institute of Plant Protection, Chinese Academy of Agricultural Sciences, Beijing 100193, China; zzhou@zju.edu.cn; 2 London Research and Development Centre, Agriculture and Agri-Food Canada, London, ON N5V 4T3, Canada; jonathan.griffiths@agr.gc.ca

It is with profound sadness and a deep sense of loss that we mourn the passing of Professor Andrew Otis Jackson on 6 July 2025. Andy was a towering figure in plant virology, whose pioneering research on plant RNA viruses, particularly hordeiviruses and rhabdoviruses, fundamentally reshaped our understanding of viral replication, movement, and pathogenesis. This tribute from a few of the countless virologists he either directly mentored, or supported and inspired, aims to honor his immense scientific legacy. Just as importantly, this Special Issue is meant to celebrate his unparalleled generosity as a mentor, colleague, and friend. We are honored to dedicate the Special Issue “Plant Virus Research and Biotechnology-Based Resistance Strategies—In Honor of Professor Andrew Otis Jackson” to his memory.

## 1. A Pioneering Scientific Journey: The Chip on the Stream

Andy often described his career trajectory as that of “a chip floating on a stream,” a modest metaphor for a journey marked by curiosity, serendipity, and transformative discoveries [1]. Born on a farm in Enterprise, Alabama, the United States, in 1941, his early observations of nature and plant diseases seeded a lifelong passion for the biological sciences. His academic path, from a junior in college in Oklahoma guided by the supportive Dean Meraz, to his Ph.D. on cereal rust fungi at the University of Manitoba, Winnipeg, Manitoba, Canada, and finally to his pivotal postdoctoral work in virology at the University of Arizona and the University of Nebraska, set the stage for a remarkable independent career.


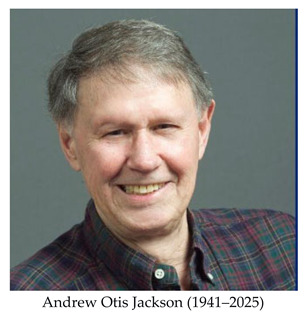


His faculty appointments at Purdue University and later at the University of California, Berkeley, catalyzed his most influential work. Andy’s lab pioneered the molecular characterization of *barley stripe mosaic virus* (BSMV), establishing it as the type member of the *Hordeivirus* genus [2,3,4,5,6,7]. His team meticulously mapped the tripartite genome of BSMV, identified the roles of the triple gene block (TGB) proteins in viral movement, and uncovered the multifunctional nature of the γb protein as an RNA silencing suppressor and pathogenesis factor. This body of work provided a foundational model for understanding virus–host interactions in monocot plants.

Simultaneously, he embarked on a decades-long quest to unravel the complexities of plant rhabdoviruses, using *sonchus yellow net virus* (SYNV) as his primary model. Overcoming immense technical challenges, his lab sequenced the entire SYNV genome, demonstrated nuclear replication—a key distinction from cytoplasmic animal-infecting counterparts—and characterized SYNV structural proteins [8,9,10]. The crowning achievement of this endeavor was the development of the first reverse genetics system for a plant negative-strand RNA virus [11,12,13,14]. This breakthrough, achieved through ingenious agroinfiltration-based strategies, allowed for the rescue of infectious recombinant SYNV, opening up entirely new avenues for studying virus morphogenesis, movement, and host adaptation [15,16]. In addition to laying the groundwork for infectious clones of other negative-strand RNA viruses including *barley yellow striate mosaic virus* and *tomato spotted wilt virus* [17,18], his pioneering system was successfully adapted for other complex plant viruses. His early exploratory work thus provided an indispensable foundation for subsequent advances in the field, cementing his status as a visionary in the field.

## 2. The Heart of a Mentor: Nurturing Generations of Scientists

While his scientific publications form an enduring legacy, Andy’s most profound impact was perhaps on the people he guided. He was a mentor in the truest sense, investing not just in projects, but in the personal and professional development of every student, postdoc, and colleague who crossed his path.

His approach to mentorship was holistic. He was deeply committed to imparting scientific knowledge and cultivating rigorous experimental habits. In the lab, he emphasized the importance of meticulous note-taking, a practice he admired in his first graduate student, Gary Gustafson, and encouraged in all who followed. He fostered critical academic thinking by engaging in lively, impromptu discussions, challenging assumptions, and pushing to design elegant, definitive experiments.

The process of manuscript and grant writing under his guidance was a masterclass in scientific communication. Andy would provide repeated, thorough, and critically constructive revisions. He had an uncanny ability to identify logical gaps and strengthen narratives. For early-career scientists, facing the anxiety of a manuscript submission or a grant decision, his guidance was a steadying force. He normalized this “feeling of insecurity,” reassuring that it was a universal experience and, more importantly, a driving force that “prompts you to continuously correct, refine, and discover new questions.” This perspective transformed professional vulnerability into a catalyst for growth.

His support extended far beyond the lab bench. Andy was a powerful advocate, providing strong, compelling recommendation letters for postdoctoral positions and faculty jobs. He took a genuine interest in the career planning for these that need his help, particularly young plant virologists and pathologists and graduate students, offering sage advice on navigating the academic landscape. His generosity knew no borders: he facilitated collaborations, secured funding for young Chinese scientists to work in his Berkeley lab and to attend key international conferences, and even offered personal financial and life assistance to those in need. He believed in paying forward the mentorship he had received from figures like Dean Meraz, Myron Brakke, and Albert Siegel.

Andy’s love for people was evident in his joy for conversation and friendship. He was a natural storyteller and during his lectures he would often weave in “relevant personal interest stories” to invigorate the material and captivate his audience. This approach was especially impactful in his interactions with Chinese graduate students. At China Agricultural University and Zhejiang University, where he spent extended periods teaching and mentoring, Andy was known for his heuristic and encouraging teaching style. He did not just impart knowledge; he asked probing questions that stimulated critical thinking and gently guided his students to discover solutions on their own. He made a point of celebrating their progress, building their confidence as much as their expertise. His connection to China was particularly special. With a 10-year visa in hand, he had eagerly planned several return trips before 2028 to continue these fruitful academic exchanges and, as he put it, “to see old friends.” This collaborative spirit, rooted in genuine mentorship and mutual respect, left an indelible mark on the field of plant virology in China and inspired a new generation of scientists.

## 3. Concluding Remarks

In his autobiographical reflection [1], Andy wrote, “These mentoring activities have been gratifying for me as they provide a feeling of reciprocation to those who were so critical to encouraging my early career activities.” This sentiment captures the essence of the man. He built not just a research program, but a global family of scientists who cherished his wisdom, his warmth, and his unwavering belief in their potential.

Looking back, the “chip” that was Andrew Otis Jackson did not merely float passively; it navigated with purpose, charting new scientific territories and guiding countless other chips along the stream. His scientific rigor, his innovative spirit, and his profound humanity have left a blueprint for excellence and mentorship that will inspire generations to come.

Thank you, Andy, for everything. You will be profoundly missed, but your legacy will continue to flourish in the work of all those you touched.

## Data Availability

Not applicable.
